# Sigmoid Volvulus in Pregnancy Assessed by Contrast-Enhanced Computed Tomography Scanning

**DOI:** 10.1155/2021/6692483

**Published:** 2021-03-03

**Authors:** Toshiaki Watanabe, Tadatsugu Kinjo, Yoshino Kinjyo, Hayase Nitta, Hitoshi Masamoto, Keiko Mekaru, Yoichi Aoki

**Affiliations:** Department of Obstetrics and Gynecology, Graduate School of Medicine, University of the Ryukyus, 207 Uehara Nishihara, Okinawa 903-0215, Japan

## Abstract

Sigmoid volvulus requires urgent treatment, and it is particularly rare among pregnant women without a history of laparotomy. A delay in diagnosis may lead to serious consequences for the mother and fetus, and a rapid diagnosis and treatment in this setting is essential. The patient was a 19-year-old primiparous woman. She complained of repeated exacerbations and remissions of abrupt lower abdominal pain for the past 2 days and was transported to our hospital at 33 weeks of gestation. Ultrasonography revealed no placental thickening, and maternal bowel dilation was difficult to identify. Plain abdominal X-ray showed a dilated colon on the left side of the abdomen. Contrast-enhanced CT scan of the abdomen revealed a volvulus on the dorsal side of the uterus. The proximal end of the transverse to sigmoid colon was markedly dilated, and the distal end was collapsed. The elevated lactate level on blood gas analysis suggested intestinal ischemia. She was suspected of having a sigmoid volvulus at 33 weeks and 3 days of gestation. We decided to perform a cesarean section to secure the operative field for an intestinal resection following delivery. A male weighing 1840 g with Apgar scores 8/8 was delivered. The sigmoid colon was approximately 80 cm in length. A 360-degree clockwise rotation of was observed with a very distended but viable sigmoid loop. Following reduction of the volvulus, the sigmoid colon was fixed to the left side of the peritoneum. The mother had an uneventful postoperative course, and the infant was discharged without any sequelae. This case demonstrates two important lessons. First, sigmoid volvulus can occur in pregnant women even if they never had a laparotomy. Second, abdominal contrast-enhanced CT is useful for rapid diagnostic and treatment decisions relative to this pathology.

## 1. Introduction

Bowel obstruction caused by sigmoid volvulus requires urgent treatment, and it is more common among the elderly and patients with neuropsychiatric disorders [1]. It is particularly rare among pregnant women without a history of laparotomy [2–5]. A delay in diagnosis may lead to serious consequences for the mother and fetus, and a rapid diagnosis and treatment in this setting is essential.

Physicians are reluctant to perform a computed tomography (CT) scan, which is useful for diagnosis and evaluation of intestinal ischemia, on pregnant women for fear of exposing the fetus to radiation. We report a case of sigmoid volvulus diagnosed by CT scan at 33-week gestation in which the mother underwent an emergency cesarean section followed by release of the volvulus with a good outcome.

## 2. Case Report

**Figure 1:**
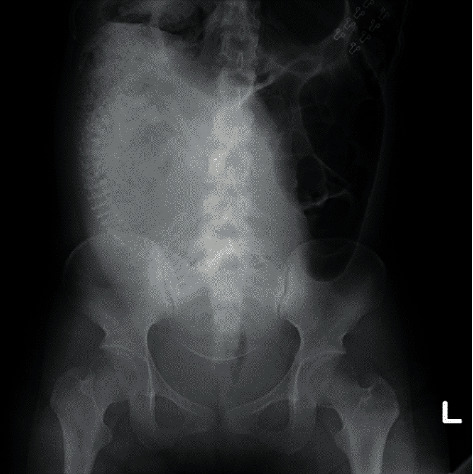
Plain abdominal radiograph showing left-sided dilated bowel.

The patient was a 19-year-old primiparous woman. Her pregnancy was uneventful until 33-week gestation when she visited her obstetrician complaining of repeated exacerbations and remissions of abrupt lower abdominal pain for the past 2 days. She received 66.7 *μ*g/min of ritodorine hydrochloride, 1000 mg of acetaminophen, and 15 mg of pentazocine, intravenously for the symptoms. A plain abdominal CT scan revealed a suspected sigmoid volvulus, and she was transported to our hospital. On admission, the abdomen was protruding due to the pregnant uterus, but there was no tenderness or rebound pain. Laboratory data were as follows: WBC 10.4 × 10^3^/*μ*L, AST 11 IU/L, ALT 9 IU/L, LDH 120 IU/L, and CRP 0.22 mg/dL. A blood gas analysis was pH 7.271, pCO_2_ 52.9 mmHg, HCO_3-_13.8 mEq/L, lactate 6.7 mmol/L, and BE -11.7 mEq/L. The fetal heart rate tracing was reassuring pattern with a baseline of 145 bpm and irregular, weak uterine contractions. Ultrasonography revealed no placental thickening, and maternal bowel dilation was difficult to identify. Plain abdominal X-ray showed a dilated colon on the left side of the abdomen (Figure 1).

**Figure 2:**
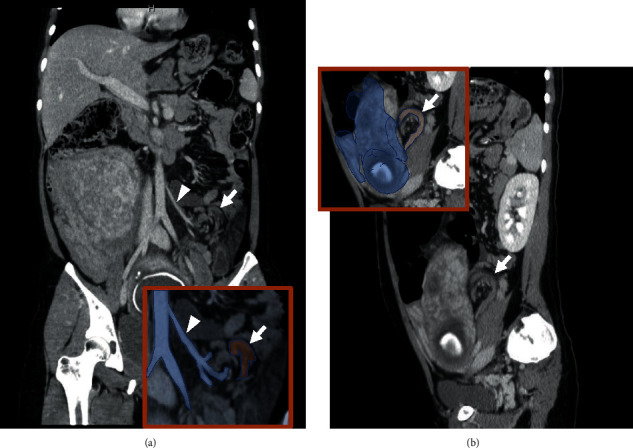
(a) Abdominal contrast-enhanced computed tomography (CT) (coronal view) with sigmoidal torsion (arrow) at the periphery of the inferior mesenteric artery (arrowhead). (b) Abdominal contrast-enhanced CT (sagittal view)—a portion of the torsion is located on the dorsal side of the uterus (arrow).

Contrast-enhanced CT was needed to confirm the diagnosis and determine the treatment plan. Contrast-enhanced CT scan of the abdomen revealed a volvulus on the dorsal side of the uterus. The proximal end of the transverse to sigmoid colon was markedly dilated, and the distal end was collapsed. There was no area of poor contrast uptake within the bowel wall (Figures 2(a) and 2(b)), and there was ascites which had not been seen on a plain abdominal radiograph 3 hours earlier. We did not apply a low-dose CT protocol, but it was nighttime and we were not able to do it in our institute. CT parameters were as follows: pitch 55, 120 kilovoltage (kV), and 400-600 (Auto) mA. Estimated fetal radiation was 23.12 mGy. We administered 100 ml of ioversol, and portal phase was acquired, because we thought that the diagnostic benefit was judged to outweigh the risk.

**Figure 3:**
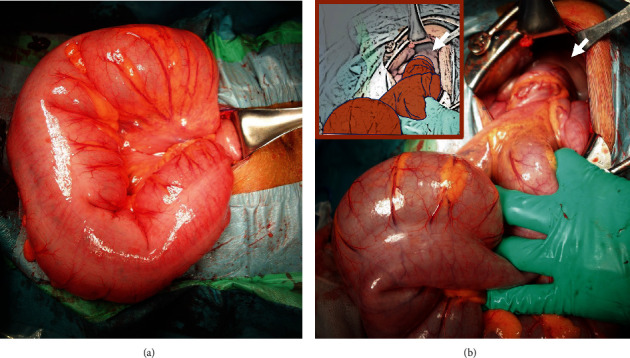
(a) Intraoperative photograph—approximately 80 cm of the sigmoid colon is dilated. (b) Intraoperative finding—360-degree clockwise rotation of the sigmoid colon (arrow).

The elevated lactate level on blood gas analysis suggested intestinal ischemia. The woman was suspected of having a sigmoid volvulus at 33 weeks and 3 days of gestation. Because the site of the volvulus was located on the dorsal side of the uterus, we decided to perform a cesarean section to secure the operative field for an intestinal resection following delivery. A male weighing 1840 g with Apgar scores 8/8 was delivered by cesarean section. The sigmoid colon was approximately 80 cm in length and highly mobile. A 360-degree clockwise rotation of the colon was observed with a very distended but viable sigmoid loop. No intestinal ischemia or necrosis was observed (Figures 3(a) and 3(b)). Following reduction of the volvulus, the sigmoid colon was fixed to the left side of the peritoneum. The mother had an uneventful postoperative course and was discharged after 7 days. The infant was cared for in the neonatal intensive care unit and was discharged without any sequelae.

## 3. Discussion

Sigmoid volvulus during pregnancy was first reported by Braun in 1885 when an autopsy of the mother confirmed the finding. A review of the literature found 112 cases of intestinal volvulus complicating pregnancy, most commonly involving the sigmoid colon (*n* = 52, 46%) [6]. Sigmoid volvulus is a rare condition, and to date, approximately 120 cases have been reported [2–5]. The pathophysiology of pregnancy, including gestational anatomical and physiological changes such as increased uterine drainage, progesterone-induced bowel relaxation, decreased peristalsis, and retention of feces, may be a factor in the pathogenesis of sigmoid volvulus, with it occurring more commonly in pregnant than in nonpregnant woman [1, 5]. The mechanism of volvulus in pregnancy appears to be an abnormally mobile sigmoid colon due to the enlarged uterus, resulting in the colon being pushed out of the pelvis and rotating around its fixation point [1]. In our case, the intestinal tract was likely extremely long and overstretched by about 80 cm, making it highly mobile and prone to torsion.

Maternal mortality from sigmoid volvulus during pregnancy has been reported to be 5% when associated with viable bowel; however, it increases to over 50% if there is gangrene or the colon perforates [3, 4]. Fetal mortality is about 40%, and a reduction in placental blood flow due to hypovolemia or decreased pelvic blood flow due to bulky sigmoid dilatation could be the causes of fetal death [5]. A delay in diagnosis may lead to serious consequences for the mother and fetus due to bowel infarction resulting in electrolyte unbalance, metabolic acidosis, septic shock, and multiple organ failure. It is essential to make a rapid diagnosis in this setting. The advantage of using contrast media is that it shows the running of blood vessels and ischemia in the intestinal wall, leading to the subsequent treatment. Actually, the immediate surgery could be performed after the CT scan.

Abdominal contrast-enhanced CT, which is often not considered over concerns for fetal radiation exposure, is useful for diagnostic and treatment decisions. Even if dilated bowel is confirmed by abdominal plain radiography, it is difficult to determine the exact site of the volvulus. CT can provide a large amount of information in a short period of time, nd it is useful for definitive diagnosis [7, 8]. Diagnostic radiation during this type of exam usually results in fetal exposure of less than 50 mGy, and the risk of developing malformations, developmental delay, malignant tumors, or genetic diseases is uncommon from this degree of exposure during pregnancy [8, 9]. There was a report of transient hypothyroidism in a neonate who was exposed to contrast media from fetal amniography using both water-soluble and oil-based media at 35-week gestation. Guidelines recommend monitoring of thyroid function in the newborn following birth if the mother is given contrast media during pregnancy [9, 10]. In our case, the thyroid function of the infant was determined shortly after delivery and it was normal.

The initial choice of treatment for sigmoid volvulus is noninvasive endoscopic revision in the absence of bowel perforation or findings of peritonitis [4]. However, in cases of intestinal perforation and peritonitis or in cases where endoscopic repair is unsuccessful, emergency laparotomy or bowel resection is generally performed [2, 3, 5]. Additionally, two issues specific to pregnant women, changes in anatomical structure during pregnancy and fetal prognosis, should be considered. The incidence of sigmoid volvulus during pregnancy by trimester was reported to be 6%, 19%, 54%, and 21% in the 1^st^, 2^nd^, and 3^rd^ trimesters and postpartum period, respectively [6]. A cesarean section often precedes bowel repair in the 3^rd^ trimester, because intact survival can be expected. In the 1^st^ trimester, the only option is a volvulus release or bowel resection, after explaining the possibility of miscarriage. In the 2^nd^ trimester, the optimum procedure is sometimes difficult to determine, but the mother's life is the priority at that point, and if the pregnant uterus affects treatment, delivery must be considered. There is no reason to deliver the infant if the bowel is repaired, and it is possible to continue the pregnancy due to strangulated bowel obstruction; fetal mortality is relatively high from the effects of respiratory and circulatory failure, sepsis, and electrolyte abnormalities [3–5]. Fetal well-being should be carefully monitored if the pregnancy is able to continue.

In summary, this case demonstrates two important lessons. First, sigmoid volvulus can occur in pregnant women even if they never had a laparotomy. Second, abdominal contrast-enhanced CT, which is often not used over concerns for fetal radiation exposure, is useful for rapid diagnostic and treatment decisions relative to this pathology.
